# Guanabenz Interferes with ER Stress and Exerts Protective Effects in Cardiac Myocytes

**DOI:** 10.1371/journal.pone.0098893

**Published:** 2014-06-03

**Authors:** Christiane Neuber, June Uebeler, Thomas Schulze, Hannieh Sotoud, Ali El-Armouche, Thomas Eschenhagen

**Affiliations:** 1 Department of Experimental Pharmacology and Toxicology, University Medical Center Hamburg-Eppendorf, Hamburg, Germany; 2 DZHK (German Center for Cardiovascular Research), partner site, Hamburg/Kiel/Luebeck, Germany; 3 Department of Pharmacology, University Medical Center Goettingen, Goettingen, Germany; 4 DZHK (German Center for Cardiovascular Research), partner site Goettingen, Germany; 5 Department of Pharmacology, University of Technology Dresden, Dresden, Germany; University of Torino, Italy

## Abstract

Endoplasmic reticulum (ER) stress has been implicated in a variety of cardiovascular diseases. During ER stress, disruption of the complex of protein phosphatase 1 regulatory subunit 15A and catalytic subunit of protein phosphatase 1 by the small molecule guanabenz (antihypertensive, α_2_-adrenoceptor agonist) and subsequent inhibition of stress-induced dephosphorylation of eukaryotic translation initiation factor 2α (eIF2α) results in prolonged eIF2α phosphorylation, inhibition of protein synthesis and protection from ER stress. In this study we assessed whether guanabenz protects against ER stress in cardiac myocytes and affects the function of 3 dimensional engineered heart tissue (EHT). We utilized neonatal rat cardiac myocytes for the assessment of cell viability and activation of ER stress-signalling pathways and EHT for functional analysis. (i) Tunicamycin induced ER stress as measured by increased mRNA and protein levels of glucose-regulated protein 78 kDa, P-eIF2α, activating transcription factor 4, C/EBP homologous protein, and cell death. (ii) Guanabenz had no measurable effect alone, but antagonized the effects of tunicamycin on ER stress markers. (iii) Tunicamycin and other known inducers of ER stress (hydrogen peroxide, doxorubicin, thapsigargin) induced cardiac myocyte death, and this was antagonized by guanabenz in a concentration- and time-dependent manner. (iv) ER stressors also induced acute or delayed contractile dysfunction in spontaneously beating EHTs and this was, with the notable exception of relaxation deficits under thapsigargin, not significantly affected by guanabenz. The data confirm that guanabenz interferes with ER stress-signalling and has protective effects on cell survival. Data show for the first time that this concept extends to cardiac myocytes. The modest protection in EHTs points to more complex mechanisms of force regulation in intact functional heart muscle.

## Introduction

Proper endoplasmic reticulum (ER) function is essential for the maintenance of cellular processes such as protein assembling, calcium homeostasis and lipid biosynthesis [1]. The ER is highly sensitive to changing environmental conditions. Alterations of ER homeostasis disrupt correct protein folding and lead to accumulation of unfolded and misfolded proteins within the cell. To cope with ER stress conditions the cell initiates several signaling cascades known as the unfolded protein response (UPR). Three transmembrane proteins sense the protein folding status in the ER lumen: inositol-requiring protein-1 (IRE1), activating transcription factor-6 (ATF6) and protein kinase RNA (PKR)-like ER kinase (PERK). These UPR sensors are in an inactive state while they are coupled to glucose-regulated protein 78 kDa (GRP78). In case of ER stress and accumulation of unfolded proteins, GRP78 is released allowing oligomerization and subsequent activation of the typical sensors. This results in immediate (i) transcriptional up-regulation of ER-chaperones and folding enzymes to correct protein folding, (ii) translational attenuation to reduce total protein load of the ER, and (iii) activation of the ER-associated protein degradation (ERAD) pathway to protect from misfolded proteins [1]. In particular, activation of PERK decreases global protein synthesis rate by phosphorylating the α-subunit of eukaryotic translation initiation factor-2 (eIF2α). PERK simultaneously activates the transcription factor ATF4, which in turn controls expression of another transcription factor C/EBP homologous protein (CHOP) and consequently the further expression of GADD34 [2]–[4]. The growth arrest and DNA damage gene (GADD)34 is also known as PPPP1R15A, a regulatory subunit of the catalytic subunit of protein phosphatase 1 (PP1c). It participates in a negative feedback loop that terminates UPR signaling. Accordingly, stress-induced expression of GADD34 mediates dephosphorylation of eIF2α via recruitment of PP1c and thereby allows recovery of protein synthesis [5]–[7].

Pharmacological inhibition of GADD34 by guanabenz, an α*_2_*-adrenergic receptor agonist formerly used in the treatment of hypertension [8], increased survival of human cells exposed to different ER stressors [9]. Guanabenz binds directly to the regulatory subunit of PP1c and thereby disrupts the PPPP1R15A-PP1c complex. Consequently, guanabenz inhibits stress-induced dephosphorylation of eIF2α resulting in prolonged eIF2α phosphorylation and inhibition of protein synthesis in stressed cells. This could protect cells from further accumulation of misfolded proteins and their toxic effects [10].

This study assessed whether guanabenz also had protective effects against detrimental accumulation of misfolded proteins in cardiac myocytes and on the functional behavior of 3-dimensional engineered heart tissue (EHT). This would be relevant given that ER-stress or UPR failure have been implicated in a variety of diseases [11], [12] including cardiac hypertrophy, heart failure, atherosclerosis and ischemic heart disease [13]–[15].

## Materials and Methods

### Cardiomyocyte culture and assessment of cell viability

Neonatal rat cardiac myocytes (NRCM) were isolated from 1–3 day old neonates (Wistar and Lewis rats) as described earlier [16]. Experimental procedures were reviewed and approved by Ethics Committee, University of Hamburg. NRCM were cultured in Minimal Essential Medium (MEM) supplemented with 10% fetal calf serum (FCS), 1% penicillin/streptomycin and 1 mM 5′-brom-2′-deoxyuridin (BrdU). For assessment of cell viability cells were plated in 96-well plates at a density of 30,000 cells/well and for molecular biological analysis in 12-well plates at a density of 330,000 cells/well. Cells were maintained at 37 °C in 7% CO_2_ atmosphere and were in minimum cultured for one week before starting an experiment. One day before each experiment medium was replaced with fresh medium.

To assess cell viability ER stress was elicited by addition of fresh media containing tunicamycin (Sigma-Aldrich), hydrogen peroxide (H_2_O_2_, Merck), thapsigargin (Sigma-Aldrich) or doxorubicin (Sigma-Aldrich) in the specified concentrations. Guanabenz (Tocris) was always freshly dissolved in water and added as indicated. Cell viability was determined by using Cell viability Counting Kit-8 (Dojindo) according to the manufacturer's protocol. The water-soluble tetrazolium salt WST-8 [2-(2-methoxy-4-nitrophenyl)-3-(4-nitrophenyl)-5-(2.4-disulfophenyl)-2H-tetrazolium] is reduced by dehydrogenase activities in living cells into yellow-colored formazan, which is soluble in culture media. Absorbance was measured at 450 nm using a microplate reader (Tecan Safire 2). The amount of generated formazan is directly proportional to the number of living cells.

### Engineered heart tissue and analysis

Fibrin-based engineered heart tissues (EHT) from neonatal rat heart cells were generated and cultured as previously described [17]. Briefly, for each EHT, a 100 µl-reconstitution mix containing 4.1×10^5^ cells/EHT, bovine fibrinogen, aprotinin and Dulbecco's Modified Eagle Medium (DMEM) was mixed with 3 µl thrombin and pipetted around two elastic silicone posts. EHTs were cultured up to 21 days in culture medium (DMEM, 10% horse serum, 2% chick embryo extract, 1% penicillin/streptomycin, insulin 10 µg/ml, 33 µg/ml aprotinin). Contraction measurements were performed by video optical recording as previously described [17]. Average force, frequency, contraction and relaxation times were calculated from the recorded contractions by an algorithm that takes into account the elastic properties of the silicone posts. EHTs represent 3-dimensional heart tissue-like structures that exhibit spontaneous, regular, and synchronous beating and allow measurement of contractile force under isometric conditions.

### Quantitative real-time RT-PCR

Expression levels of mRNAs were determined by quantitative reverse transcriptase-polymerase chain reaction (qRT-PCR) [18]. Total RNA was isolated from NRCM with TRIzol (Invitrogen) and about 200 ng total RNA was reversely transcribed with High Capacity cDNA Reverse Transcription Kit (Applied Biosystems) according to manufacturer protocols. Quantitative RT-PCR was performed by using specific primers against the following rat target genes ATF4 (forward: 5'-ACC GGC AAG GAG GAT GCC TT-3', reverse: 5'- AGC TCA TCT GGC ATG GTT TC-3', transcript size: 119 bp), CHOP (forward: 5'-GCA GCT GAG TCT CTG CCT TT-3', reverse: 5'-GGG ATG CAG GGT CAA GAG TA-3', transcript size: 175 bp), CREP (forward: 5'-GAC AAT TGT CCA GGC TGT GG-3', reverse: 5'-GCA TCC ATC CCT TGC AAA TTC-3', transcript size: 161 bp), GADD34 (forward: 5'- CTG AAG GGT AGA AAG GTG CAC-3', reverse: 5'-CTT CGA TCT CGT GCA AAC TG-3', transcript size: 118 bp), GRP78 (forward: 5'-CAT CAA TGA GCC AAC AGC AG-3', reverse: 5'-CAT TAG TGG CCA CCA CTT CA-3', transcript size: 152 bp) and Maxima SYBR Green/ROX qPCR Master Mix (Thermo Scientific) with the ABIPrism 7900 Sequence Detection System (Applied Biosystems).

### Western Blot Analysis

Immunoblot analyses were performed as described earlier [16]. Membranes were incubated with the following primary antibodies against ATF4 (1∶200, Santa Cruz, pAb), CHOP (1∶1000, Cell Signaling, mAb), eIF2α (1∶1000, Cell Signaling, pAb), phoshpho-eIF2α (1∶1000, Cell Signaling, pAb), GRP78 (1∶1000, Cell Signaling, pAb), eIF4 (1∶1000, Cell Signaling, pAb), IRE1α (1∶1000, Cell Signaling, pAb), α-actinin (1∶1000, Sigma-Aldrich, mAb), calsequestrin (1∶2500, Dianova, pAb) and with secondary antibodies anti-rabbit IgG POX (1∶5000, Sigma-Aldrich) and anti-mouse IgG POX (1∶5000, Sigma-Aldrich).

### Statistical analysis

All statistical tests were performed in GraphPad Prism version 5.02. In detail, one-way ANOVA and Dunnett's multiple comparison post-test (to compare to control condition) or two-way ANOVA and Bonferroni's multiple comparison post-test (to compare all groups) were used for more than two groups. Average data are presented as mean ± SEM. Differences were considered significant when p<0.05. P-values are displayed graphically as follows: *P<0.05, **P<0.01, ***P<0.001.

## Results

### Treatment of neonatal rat cardiomyocytes with tunicamycin or guanabenz

**Figure 1:**
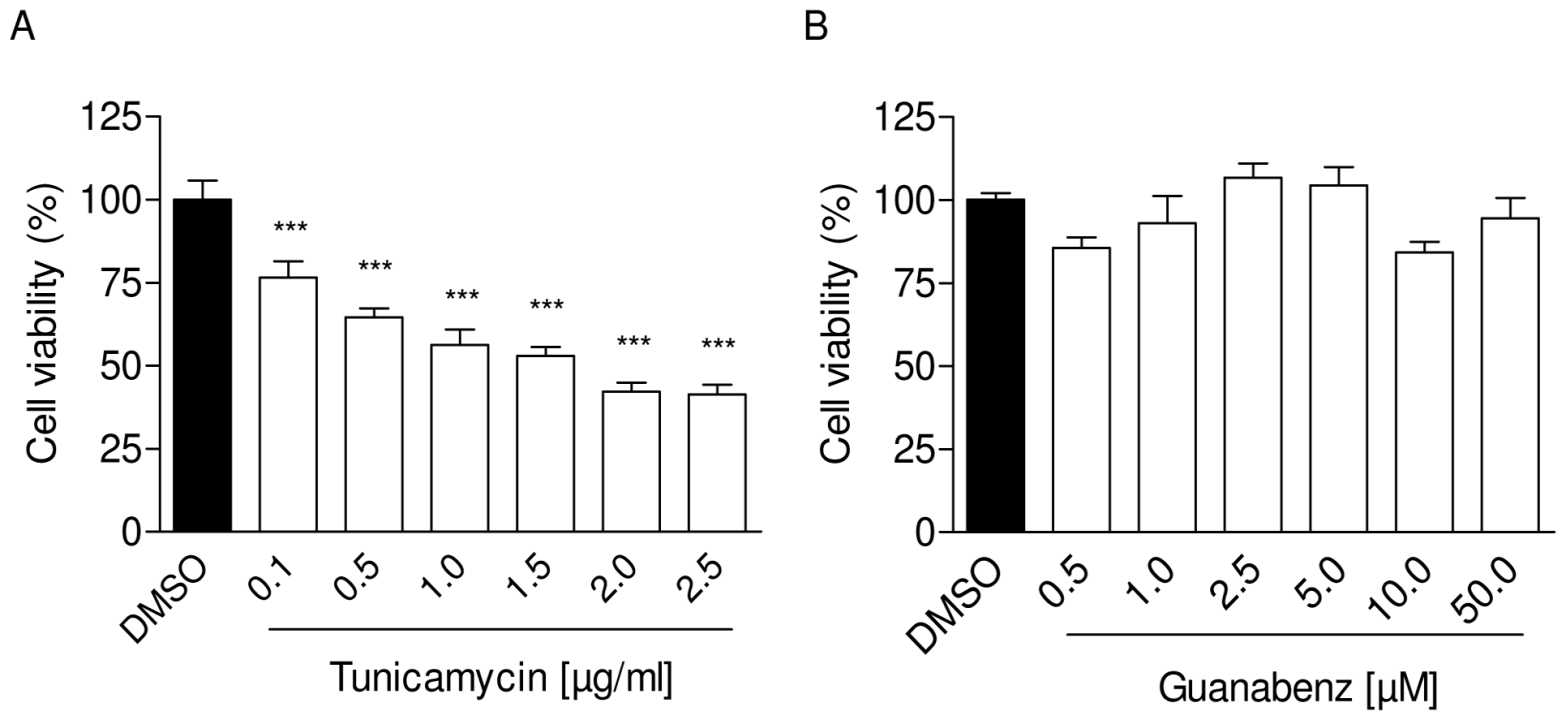
Effects of tunicamycin and guanabenz on cell viability of neonatal rat cardiomyocytes (NRCM). (A) Concentration-dependent reduction of cell viability by tunicamycin treatment for 24 hours. (B) Treatment with increasing concentrations of guanabenz for 24 hours did not affect cell viability. Data are means ± SEM (n = 8); ***P<0.0001.

**Figure 2:**
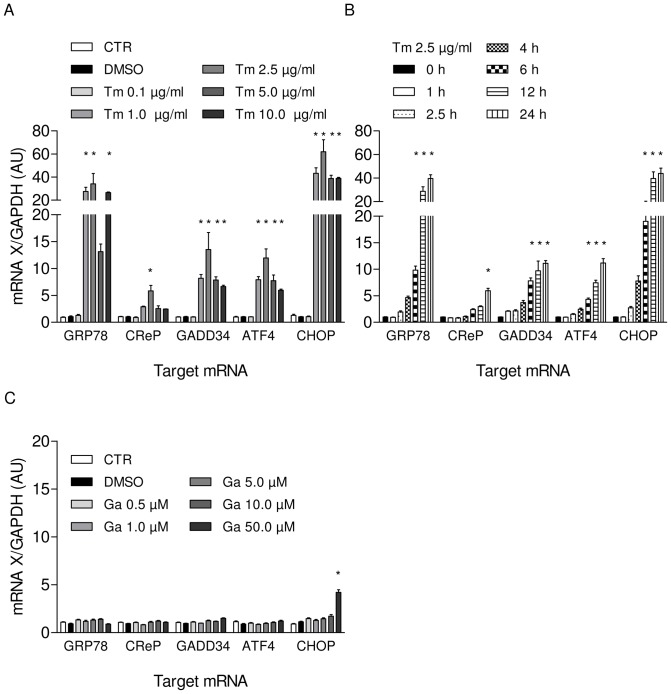
Effects of tunicamycin and guanabenz on different branches of the UPR in NRCM on the mRNA level. (A) Tunicamycin (Tm) treatment of NRCM for 12 hours increased levels of the UPR targets GRP78, GADD34, ATF4, and CHOP. (B) Time-dependent increase of UPR targets after treatment with tunicamycin (2.5 µg/ml). (C) Guanabenz (Ga) treatment of NRCM for 24 hours did not affect levels of UPR targets. Data are means ± SEM (n = 3–5); Changes of mRNA expression of ≥1.6 or ≤0.5 compared to DMSO control and a corrected P-value<0.05 were considered significant.

**Figure 3:**
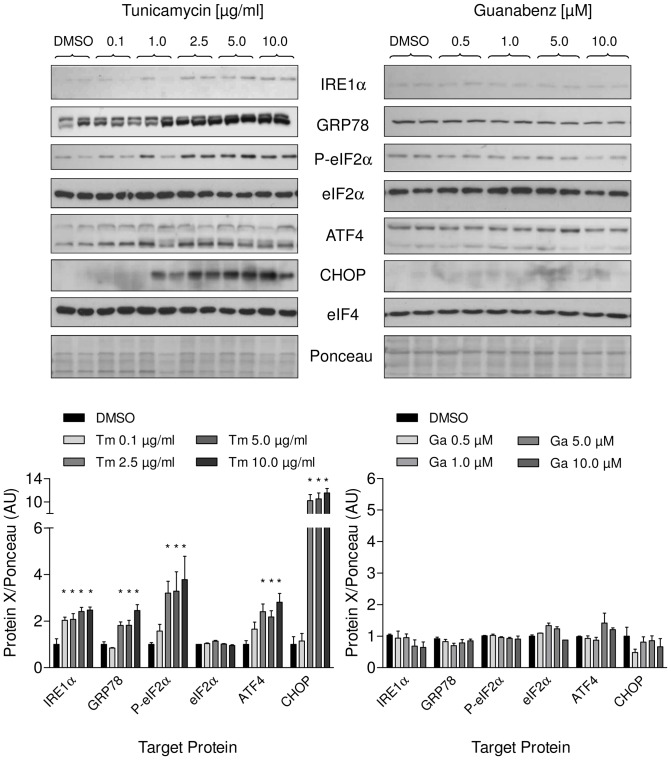
Effects of tunicamycin and guanabenz on different branches of the UPR in NRCM on the protein level. Upper panel – representative immunoblots of lysates of NRCMs treated with tunicamycin (left) or guanabenz (right) for 12 and 24 hours, respectively. Lower panel – concentration-dependent increase of UPR targets proteins after treatment with tunicamycin (Tm) or guanabenz (Ga) compared to DMSO control. Please note that the intensity of the upper unspecific band for GRP78 is caused by differences in blotting conditions (10% acrylamide/bisacrylamide gel). Data are means ± SEM (n = 3); *P<0.05.

To induce ER stress in neonatal rat cardiomyocytes (NRCM) we treated cells with increasing concentrations of tunicamycin (0.1–2.5 µg/ml) and measured cell survival after 24 hours. As expected, tunicamycin reduced cell viability in a concentration-dependent manner (Figure 1A), whereas guanabenz (0.5–50 µM) did not affect cell survival (Figure 1B). Additionally, we measured the effect of the treatment on different branches of UPR on mRNA and protein level. Tunicamycin strongly upregulated mRNA concentrations of the ER-chaperone GRP78 and activated the PERK pathway, which is reflected by the increase of the typical UPR targets ATF4, GADD34 and CHOP in a concentration- and time-dependent manner (Figure 2A and B). Accordingly, protein amounts of GRP78, ATF4 and CHOP were elevated as well (Figure 3 left panel). Increased eIF2α phosphorylation (Figure 3 left panel) indicated inhibiton of the translational machinery. Besides activation of the PERK pathway, we could detect activation of a second UPR pathway that is initiated by IRE1α resulting in concomitant elevation of its protein amount (Figure 3 left panel). The third UPR pathway that is reflected by initiation of ATF6 was not activated by tunicamycin (data not shown).

As expected, guanabenz alone (0.5–50 µM) did not affect these UPR targets, neither on mRNA (Figure 2C) or protein level nor the phosphorylation status of eIF2α (Figure 3 right panel). It also did not induce its proposed pharmacological target GADD34 or the constitutively active form CReP (Figure 2C). Taken together, the glycosylation inhibitor tunicamycin induced ER stress in neonatal rat cardiomyocytes that activated the different branches of the UPR while guanabenz alone had no effect.

### Treatment of neonatal rat cardiomyocytes with ER stressors and guanabenz

**Figure 4:**
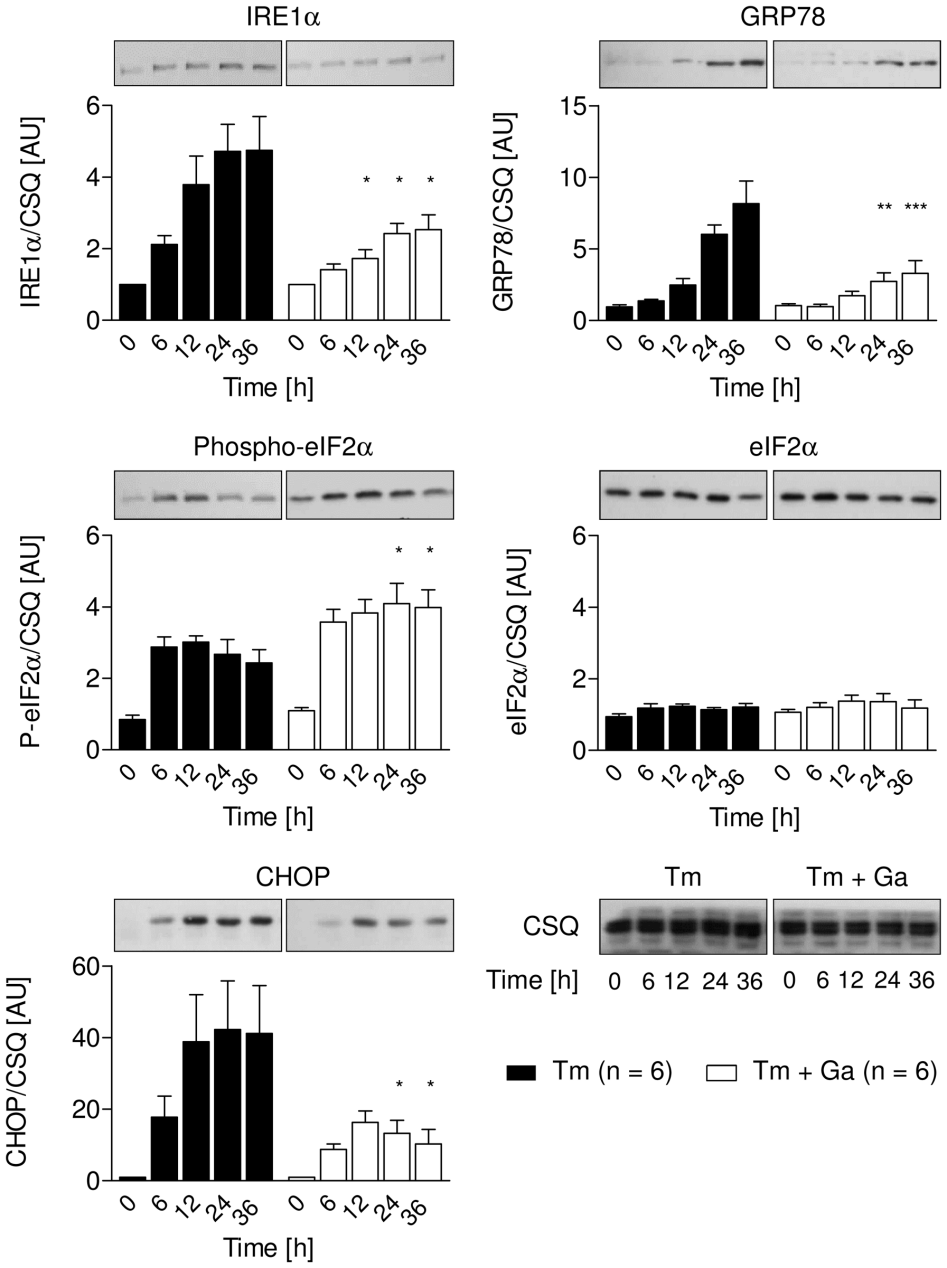
Representative immunoblots of ER stress marker proteins related to the loading control calsequestrin (CSQ) in lysates of NRCM treated with tunicamycin (Tm; 2.5 µg/ml) in the presence or absence of guanabenz (Ga; 2.5 µM) for the indicated times (0, 6, 12, 24 and 36 h). Bar graphs show densitometric and statistical analysis of the immunoblots. Data are means ± SEM (n = 6); *P<0.05 compared to the respective time points.

To examine whether guanabenz affected the different branches of the UPR in stressed cells, NRCM were treated with tunicamycin 2.5 µg/ml with or without guanabenz 2.5 µM. Protein levels of IRE1α, GRP78, CHOP as well as eIF2α phosphorylation were analysed over 36 hours (Figure 4). As seen before, tunicamycin induced the expression of the ER stress markers IRE1α, GRP78 and the pro-apoptotic protein CHOP. In addition, it increased phosphorylation of eIF2α with a maximum between 6 and 12 h and a non-significant trend for a decrease at later time points. Treatment with guanabenz markedly reduced the effect of tunicamycin on IRE1α, GRP78, CHOP levels, and significantly prolonged eIF2α phosphorylation at 24 and 36 h, compatible with the proposed mechanism of action of guanabenz.

**Figure 5:**
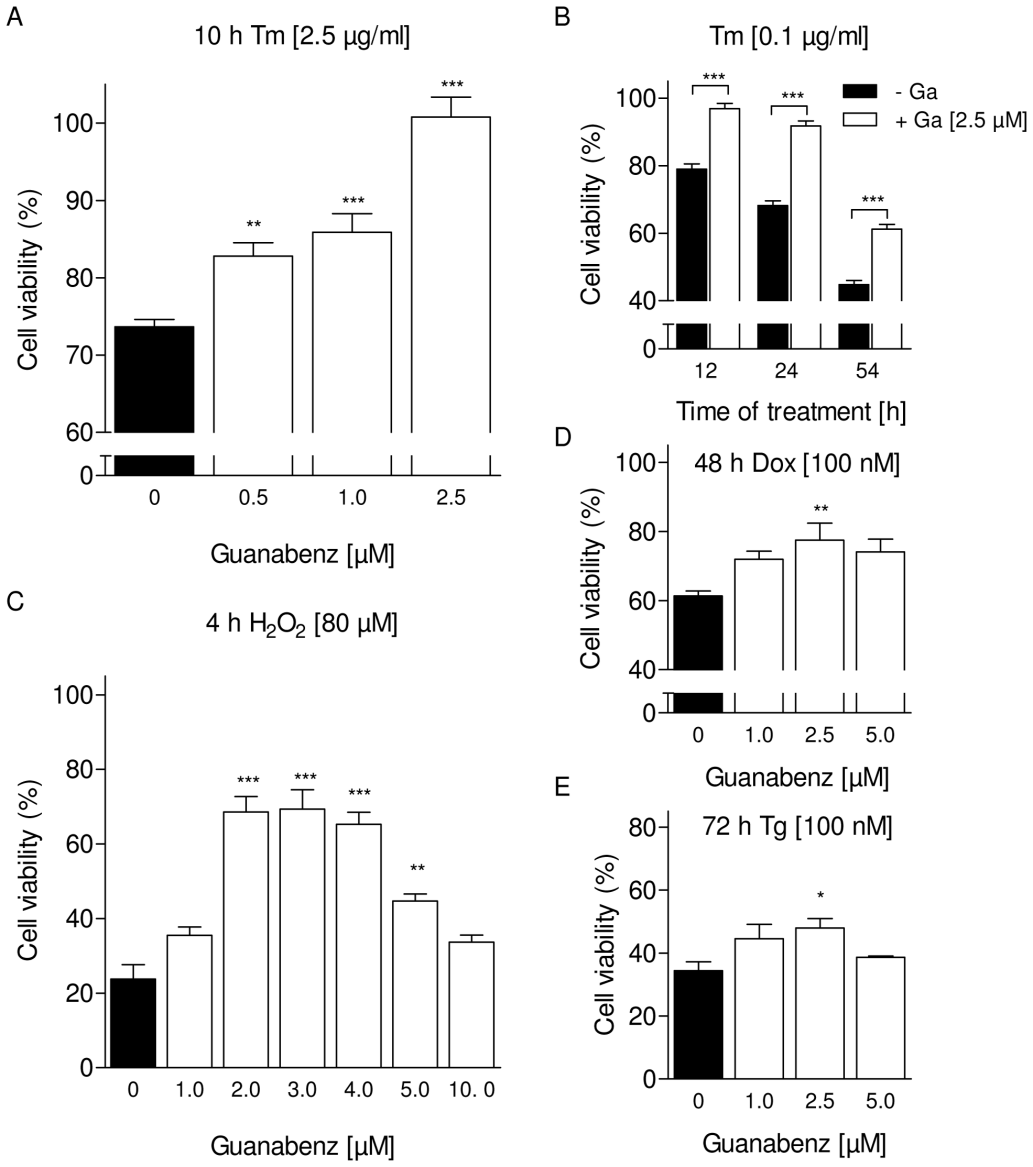
Guanabenz protected NRCM from deleterious endoplasmic reticulum stress. Values are normalized to vehicle control (100%). Data are means ± SEM. (A) Assessment of cell viability after treatment with tunicamycin (Tm; 2.5 µg/ml) and indicated concentrations of guanabenz for 10 h. (n = 8); **P<0.001; ***P<0.0001. (B) Protective effect of guanabenz (Ga; 2.5 µM) in cells treated with tunicamycin (0.1 µg/ml) for 12, 24, and 54 hours. (n = 4); ***P<0.0001. (C) Concentration-dependent protection by guanabenz against cellular stress induced by exposure to H_2_O_2_ (80 µM) for 4 hours (n = 7–14); **P<0.001; ***P<0.0001. Cell viability of NRCM treated with doxorubicin (Dox; 100 nM) for 48 hours (D) or with thapsigargin (Tg; 100 nM) for 72 hours (E), with or without indicated concentrations of guanabenz. (n = 6–7); *P<0.05 **P<0.001.

Next we assessed whether the effects of guanabenz on tunicamycin-induced ER stress extends to protection against cytotoxicity exerted by the known ER stressors tunicamycin (Figure 5A and B), hydrogen peroxide (H_2_O_2_; Figure 5C), doxorubicin (Figure 5D) or thapsigargin (Figure 5E). Like tunicamycin, these drugs are known to activate the PERK pathway in the given concentrations and have an impact on cardiomyocyte function [19]–[21]. In accordance with these data, we saw a comparable induction of UPR by hydrogen peroxide treatment (data not shown). Cytotoxicity was determined as the reduction in formazan production. Different protocols of treatment with the cytotoxic drugs were tested. All 4 compounds reduced the viability of NRCM by 25–75% compared to vehicle control. The strongest toxicity was observed with a short (4 h) pulse of a high concentration of H_2_O_2_ (80 µM). Preincubation with guanabenz promoted the survival of NRCM (Figure 5) at least at certain time points. The concentration-response relation of guanabenz was bell-shaped with a maximally effective concentration around 2–3 µM.

### Treatment of engineered heart tissue with ER stressors and guanabenz

**Figure 6:**
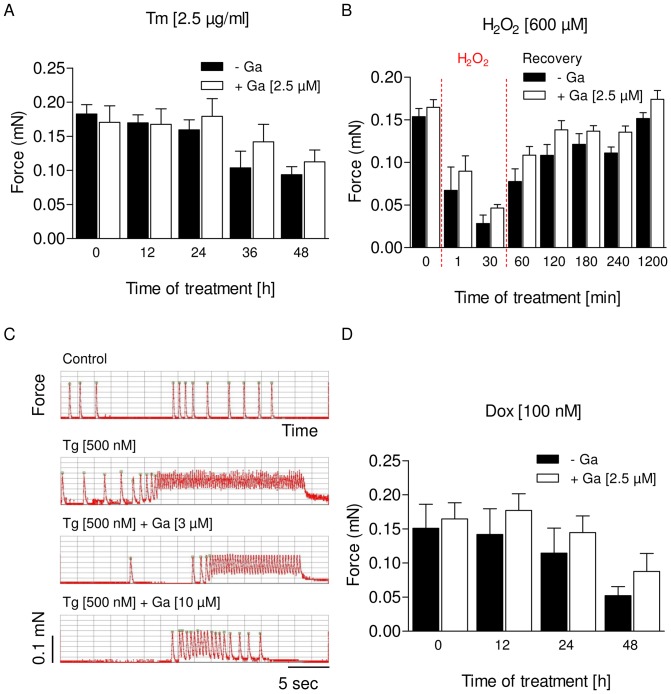
Impact of guanabenz on EHT contractility during exposure to ER stress. (A) Force development of EHTs after treatment with tunicamycin (Tm; 2.5 µg/ml) for 12, 24, 36 and 48 hours, with or without guanabenz (Ga; 2.5 µM). Pre-drug values Tm: 0.17±0.01 vs. Ga: 0.17±0.02. (B) Force development of EHTs with or without guanabenz (2.5 µM) during 30 min acute exposure to H_2_O_2_ (600 µM) and during following 20 hours recovery time. Pre-drug values Tm: 0.16±0.01 vs. Ga: 0.16±0.01. (C) Representative beating pattern of an EHT treated with thapsigargin (Tg; 500 nM) for 1 hour, with or without guanabenz (3 µM, 10 µM) compared to an untreated control EHT. (D) Force development of EHTs after treatment with doxorubicin (Dox; 100 nM) for 12, 24 and 48 hours, with or without guanabenz (2.5 µM). Pre-drug values Tm: 0.15±0.03 vs. Ga: 0.16±0.02. Data are means ± SEM (n = 4–6). All drugs showed a time-dependent effect on EHT contractility (Two-way ANOVA, row factor, P<0.05), but differences between the groups were not statistically significant.

The above results indicated a protective guanabenz effect against drug-induced ER stress and cell death in cardiac myocytes. To evaluate whether this effect also translates into preserved contractile function we employed the model of engineered heart tissue (EHT), 3-dimensional, spontaneously beating and force-developing muscle strips [17]. We incubated EHTs with equivalent concentrations of tunicamycin, thapsigargin, doxorubicin or hydrogen peroxide with and without guanabenz and measured force development over time. Preincubation with guanabenz had no effect on EHT contractility and is indicated in the respective figures as time zero (0 h). In all cases exposure to cytotoxic agents resulted in a reduction of force or in an abnormal beating pattern (Figure 6). Compared to NRCM in 2D culture the toxic effect of tunicamycin was delayed in EHTs, and higher concentrations of thapsigargin (500 nM) and hydrogen peroxide (600 µM) were needed to induce acute toxicity (reduction of peak force), indicating higher resistance to stress in the 3D culture model. Addition of guanabenz (2.5–10 µM) did not affect peak force significantly in any of these experiments, but was associated with a trend towards improved contractile function in each experiment and at each time point. A short pulse of hydrogen peroxide treatment led to a strong reduction in peak force, but EHTs fully recovered after 20 hours washout, arguing against a major role of cell death in this experiment. Accordingly, measurement of serum creatine kinase (CK) and lactate dehydrogenase (LDH) activities as markers of cardiomyocyte death showed no evidence for cell death in the EHT model (Figure S1). The SR Ca^2+^ ATPase (SERCA) inhibitor thapsigargin induced phases of very fast beating and lack of complete relaxation (Figure 6C), compatible with an inhibitory effect of the compound on Ca^2+^ reuptake into the SR in diastole. Of note, the onset of effect of thapsigargin was delayed (data not shown) and was reduced by guanabenz at 3 µM and almost completely abolished at 10 µM. This was unexpected, because guanabenz probably does not directly interfere with SR Ca^2+^ handling. It indicates that ER stress is a consequence of SERCA inhibition and participates in the disturbation of SR Ca^2+^ handling.

## Discussion

ER stress is increasingly recognized as a contributor to the pathophysiology of cardiovascular diseases, suggesting therapeutic interventions in its mechanisms as a new target for drug therapy. Yet, knowledge about exact molecular mechanisms of the UPR and signalling pathways leading to cardioprotection remains limited [14]. In principle, activation of the different UPR signalling branches is considered to protect cells from ER stress. For example, increased phosphorylation of eIF2α causes a transient reduction of global protein synthesis and thereby of the amount of new proteins that require proper folding. Prolonged eIF2α phosphorylation was associated with attenuated toxicity [9], [22]. It has been proposed that selective inhibition of GADD34, a stress-induced regulatory subunit of protein phosphatase 1 (PP1), causes selective attenuation of eIF2α dephosphorylation by PP1 in stressed cells [10], [23]–[25]. Indeed, guanabenz, a small molecule interfering with PP1-GADD34 interaction, protected cells from lethal ER stress without affecting cell function alone [9]. Here we tested whether this concept can be expanded to cardiac myocytes. The main findings were: (i) Tunicamycin induced ER stress and consequently activated several UPR signalling pathways. (ii) Guanabenz did not increase the levels of UPR targets, but antagonized the effects of tunicamycin on markers of ER stress in cardiac myocytes. (iii) Tunicamycin and other known inducers of ER stress led to cardiac myocyte death and this was concentration- and time-dependently antagonized by guanabenz. (iv) ER stressors also caused acute or delayed contractile dysfunction in EHTs, which was not significantly affected by guanabenz, with the notable exception of relaxation deficits under thapsigargin. Thus, our results in single cardiac myocytes principally support those in cultured cell lines, but modest protection in our EHT model point to a more complex mechanism in intact functional heart muscle, which may be critical for developing this approach for therapeutic purposes.

The beneficial effects of guanabenz on cell survival showed a bell-shaped concentration-dependency. For example, guanabenz in the presence of 80 µM hydrogen peroxide had no effect at 1, tripled cell survival at 2, 3 and 4 µM (∼70% compared to 23%) and had no effect at 10 µM. Similar results have been described before by Tsaytler et al. [9]. This narrow “therapeutic window” could be related to the proposed mechanism of action, but also to the interference with various targets, e.g. IRE1α or even other unknown targets. Tunicamycin treatment of cardiac myocytes induced upregulation of several UPR targets (GRP78, IRE1α, p-eIF2α, ATF4 and CHOP) including activation of GADD34 on the mRNA level, pointing indeed to activation of the PERK pathway. In our hands, guanabenz markedly reduced the effects of tunicamycin on IRE1α, GRP78, CHOP, and prolonged eIF2α phosphorylation, even though the statistics of the latter effect were only borderline significant. It is also notable that the transient nature of the increase in eIF2α phosphorylation under tunicamycin alone was not that clear in all experiments. We conclude that guanabenz mildly prolongs tunicamycin-induced phosphorylation of eIF2α in cardiac myocytes. This observation principally supports the effect in cell lines described by Tsaytler et al. [9], but was less pronounced, argueing for a more complex mechanism of guanabenz under our experimental conditions. Therefore we cannot conclude a delay of translational recovery, especially since we did not measure translation rates directly.

Besides activation of the PERK pathway, exposure to tunicamycin led to an increase in IRE1α protein amount, indicating initiation of another UPR sensor. Simplified, upon ER stress IRE1α undergoes oligomerization and activation of two distinct pathways promoting either cell survival or cell death: (i) The intrinsic endoribonuclease activity of IRE1α results in production of the transcription factor X-box binding protein (XBP)1 [1], [12], [14] that induces the expression of genes involved in protein folding and degradation of unfolded proteins to restore protein homeostasis. (ii) IRE1α mediates cell death and inflammatory signaling [26] via activation of the apoptosis signal-regulating kinase (ASK)1 [27], [28]. Both, the increase of spliced XBP1 [15] and activation of ASK1 [29] potentially contribute to the pathogenesis of heart diseases. Guanabenz clearly reduced the effect of tunicamycin on IRE1α for yet unknown reasons. It might be a direct interaction of the small molecule with elements of the IRE1α signaling pathway or a secondary effect resulting from translational repression and slowdown of global protein synthesis rate through activation of the PERK pathway. Interpretation of the data is complicated by the fact that the underlying mechanisms are not yet fully understood. On the one hand, activation of IRE1α and subsequent XBP1 splicing were shown to reduce ER stress and promote cell survival [30]–[32]. On the other hand, also inhibition of the IRE1α apoptotic downstream target ASK1 reduced cell death induced by ER stress [12], [33], [34]. For detailed information see [12], [35]. Our data show that the IRE1α pathway is activated during ER stress conditions in cardiac myocytes and reduced by concomitant guanabenz treatment. If IRE1α activation was protective, its downregulation by guanabenz may attenuate a cytoprotective effect of the drug and explain why it had only mild protective effects in cardiac myocytes and especially in EHT.

In addition to the concentration-dependency, the cytoprotective effects in cardiac myoyctes were time-dependent and varied between 4 and 72 h depending on the cytotoxic agent. This transient effect is consistent with the results obtained by Tsaytler et al. [9] and with a temporary reduction of protein synthesis by the proposed mechanism. Balanced feedback inhibition of the UPR by GADD34-mediated dephosphorylation of eIF2α seems to be essential for cell survival. Thus, sustained eIF2α phosphorylation is lethal to cells in vitro [36] and in vivo [37] as is the complete loss of eIF2α phosphorylation [38], [39]. Therapeutic interference with this process therefore likely needs to be mild and transient.

The impact of guanabenz treatment on EHTs during exposure to different ER stressors was modest and did not reach statistical significance. The only clear-cut effect was an almost complete prevention of the marked relaxation deficits induced by a high concentration of the SERCA inhibitor thapsigargin by guanabenz at 10 µM. The protective effect of guanabenz suggests that the thapsigargin-induced relaxation deficit and arrhythmic contractions are likely initiated by SERCA inhibition, but amplified by ER stress induction with subsequent disruption of intracellular calcium homeostasis, contractility, cell survival and mitochondrial integrity [20].Thus, guanabenz protects from ER stress without directly altering the effect of thapsigargin on SERCA inhibition, as relaxation time remains elevated in these EHTs. Guanabenz has numerous other effects, including anti-prion activity against various kinds of protein aggregation disorders [40], [41], agonistic effects at imidazoline receptors [42], [43], antagonistic effects at α_1_-adrenergic receptors [44] and monoamino oxidase-A [45] and probably other unprecedented effects. Effective concentrations of guanabenz in these studies ranged between nanomolar [46], [47] and millimolar [40]. In our hands protective effects were seen around 2.5 µM. At higher concentrations (30 µM) it exerted direct negative inotropic and lusitropic effects in engineered heart tissue (Figure S2), but also protected against adverse effects of high concentrations of epinephrine (data not shown). This observation may be related to yet another function of this small molecule, namely agonistic activity at the trace amine-associated receptor 1 [46] which mediates various cardiac effects of trace amines [48]-[51].

In summary, our data are principally consistent with the proposed protective effect of guanabenz via inhibition of PPP1R15A and ER stress, but also point to various other modes of action and possible targets in heart cells. This issue needs consideration when developing ER stress-directed concepts based on guanabenz for therapeutic purposes.

## Supporting Information

Figure S1 **Impact of tunicamcin treatment (Tm; 1.0 µg/ml) on EHTs compared to non-treated controls.** (A) Force development of EHTs under tunicamycin treatment after 0, 24, 48, 72, and 96 hours. (B) Measurement of lactate dehydrogenase (LDH) activity during tunicamycin treatment after 0, 48, and 96 hours. Serum creatine kinase (CK) activity was below detection level. Data are means ± SEM (n = 3); ***P<0.001 (Two-way ANOVA, Bonferroni post-test).
(TIF)

Figure S2 **Long-term effect of guanabenz treatment for 14 days (Ga; 3, 10, 30 µM) on EHT contractility compared to non-treated controls.** (A) Force development of EHTs under guanabenz treatment with the indicated concentrations. (B) Contraction time T1_80%_ (time from 20% to 100% of peak height of the force peak) and (C) relaxation time T2_80%_ (time from 100% to 20% of peak height of the force peak). Data are means ± SEM (n = 4); **P<0.01, ***P<0.001 (One-way ANOVA, Dunnett post-test).
(TIF)
