# Supplementary figures and images for: Guanabenz Interferes with ER Stress and Exerts Protective Effects in Cardiac Myocytes
Source: PLoS One. 2014 Jun 3;9(6):e98893. doi: 10.1371/journal.pone.0098893 (PMC4044035; doi:10.1371/journal.pone.0098893)

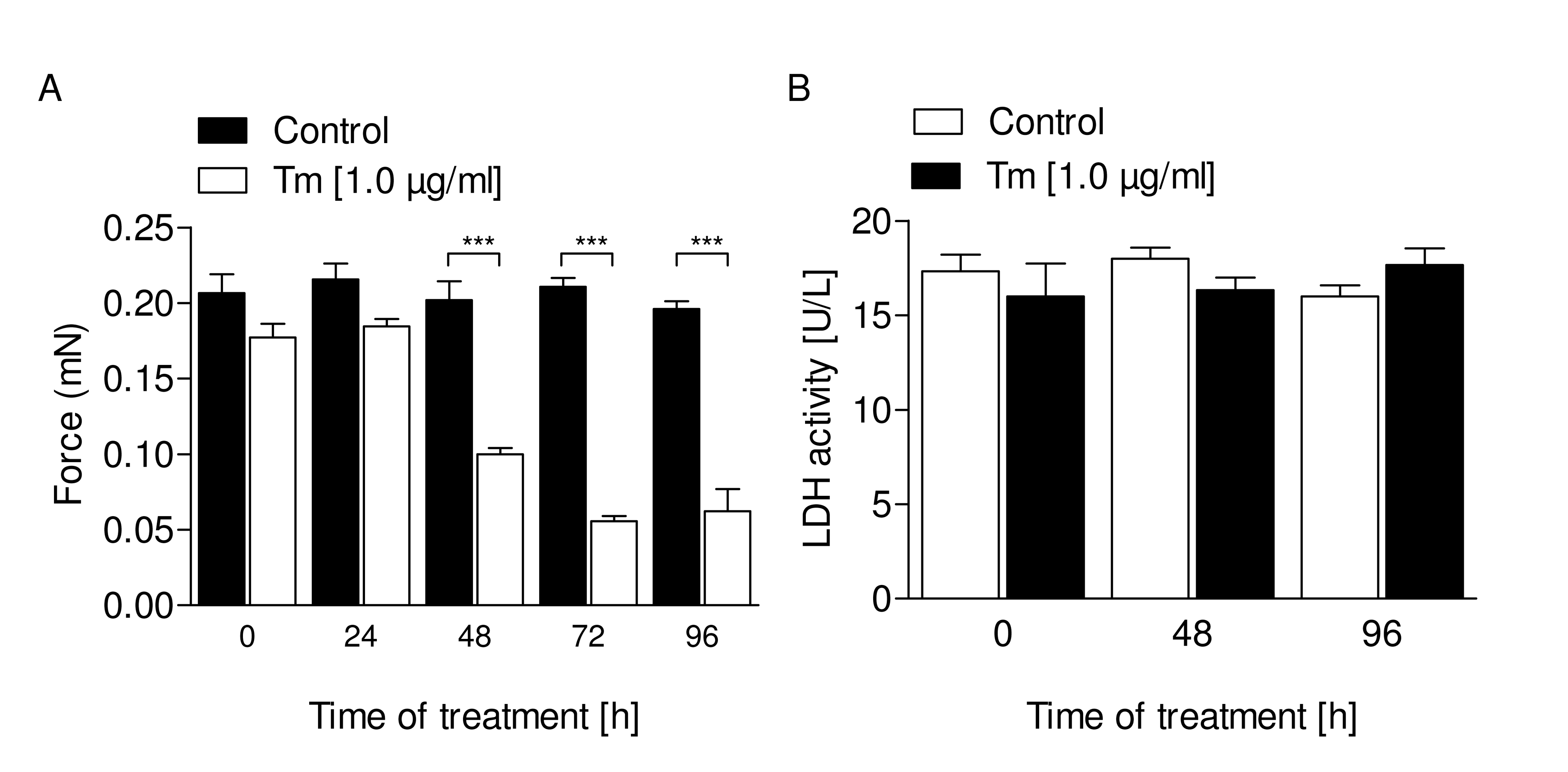

Supplement: Figure S1 — Impact of tunicamcin treatment (Tm; 1.0 µg/ml) on EHTs compared to non-treated controls. (A) Force development of EHTs under tunicamycin treatment after 0, 24, 48, 72, and 96 hours. (B) Measurement of lactate dehydrogenase (LDH) activity during tunicamycin treatment after 0, 48, and 96 hours. Serum creatine kinase (CK) activity was below detection level. Data are means ± SEM (n = 3); ***P<0.001 (Two-way ANOVA, Bonferroni post-test). (TIF) [file pone.0098893.s001.tif]

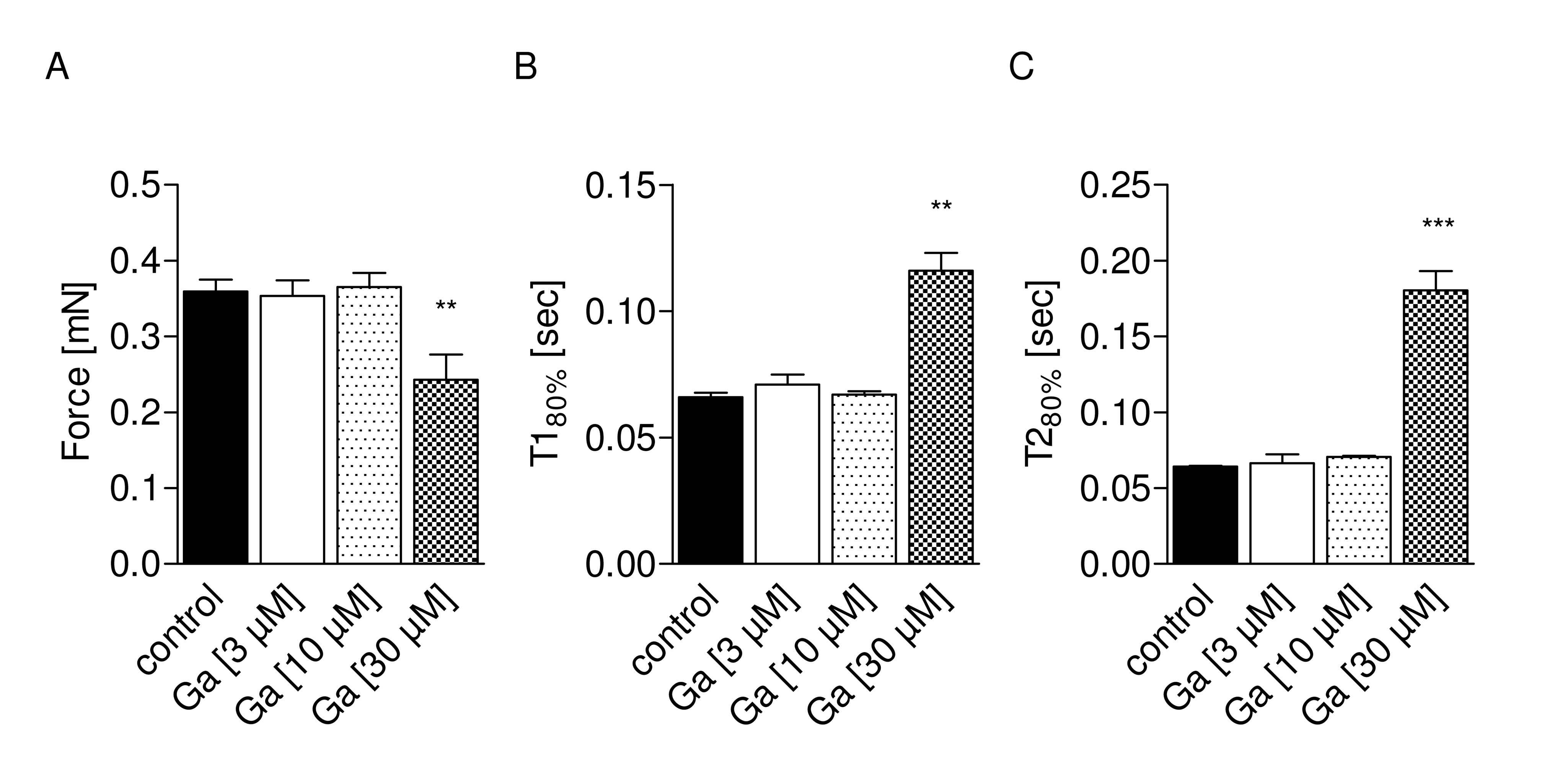

Supplement: Figure S2 — Long-term effect of guanabenz treatment for 14 days (Ga; 3, 10, 30 µM) on EHT contractility compared to non-treated controls. (A) Force development of EHTs under guanabenz treatment with the indicated concentrations. (B) Contraction time T180% (time from 20% to 100% of peak height of the force peak) and (C) relaxation time T280% (time from 100% to 20% of peak height of the force peak). Data are means ± SEM (n = 4); **P<0.01, ***P<0.001 (One-way ANOVA, Dunnett post-test). (TIF) [file pone.0098893.s002.tif]
